# Editorial: Future challenges and directions in determining allo-immunity in kidney transplantation

**DOI:** 10.3389/fimmu.2022.1013711

**Published:** 2022-08-31

**Authors:** Wai H. Lim, Julie Ho, Vasilis Kosmoliaptsis, Ruth Sapir-Pichhadze

**Affiliations:** ^1^ Department of Renal Medicine, Sir Charles Gairdner Hospital, Perth, WA, Australia; ^2^ Medical School, University of Western Australia, Perth, WA, Australia; ^3^ Department of Internal Medicine University of Manitoba, Winnipeg, MB, Canada; ^4^ Department of Immunology, University of Manitoba, Winnipeg, MB, Canada; ^5^ Transplant Manitoba Adult Kidney Program, Transplant Manitoba, Shared Health Manitoba, Winnipeg, MB, Canada; ^6^ Department of Surgery, University of Cambridge and National Institute for Health Research Cambridge Biomedical Research Centre, Addenbrooke’s Hospital, Cambridge, United Kingdom; ^7^ Blood and Transplant Research Unit in Organ Donation and Transplantation, National Institute for Health Research, University of Cambridge, Cambridge, United Kingdom; ^8^ Centre for Outcomes Research and Evaluation, Research Institute of McGill University Health Centre, Montreal, QC, Canada; ^9^ Division of Nephrology and Multi-Organ Transplant Program, Department of Medicine, McGill University, Montreal, QC, Canada

**Keywords:** allo-immune response, kidney transplant, HLA, epitope, donor specific antibodies, sensitization, HLA compatibility, immunological risk assessment

Improving long-term allograft survival remains one of the key contemporary challenges of transplantation medicine. Despite improvement in short-term kidney allograft outcomes, more than 1 in 2 kidney transplant recipients will lose their allograft within 15 years of transplantation (1). Returning to dialysis is associated with a substantial risk of death which is increased by almost 10-fold compared to patients with functioning kidney allografts (2). Maintaining a good functioning allograft over time is complex and multiple risk factors influence long-term allograft survival, ranging from organ procurement factors, post-transplant adverse events such as delayed graft function and rejection episodes, to the effects of chronic exposure to immunosuppression. To improve kidney allograft survival, both traditional and emerging potentially modifiable risk factors need to be identified.

Another equally important aspect of transplantation medicine is the assessment of sensitization status (3, 4). Pre-transplant immunological risk assessment typically involves the screening for anti-human leukocyte antigen (HLA) antibody, which can occur following prior allograft loss, infection, pregnancy and blood transfusion. Although the testing for non-HLA antibody may provide a more comprehensive profile of sensitization status pre-transplantation, the cost-benefit and cost-utility of this approach remains uncertain. Donor/recipient HLA incompatibility often increases the risk of allo-sensitization, resulting in the development of *de novo* donor specific anti-HLA antibody (dnDSA), which is strongly associated with acute rejection, premature allograft loss and reduced retransplant potential (5, 6).

The HLA system encompasses gene loci that determine tissue compatibility in organ transplantation and consequently, HLA-matching has been considered the standard triage test for immunological risk assessment for deceased donor kidney allocation worldwide allowing clinicians to modify immunosuppressive agents according to this risk (7). The HLA system is extremely polymorphic and functionally complex and in organ transplantation, this polymorphism has an important role in determining allo-immunity, including the development of acute rejection and dnDSA after transplantation. HLA-typing has evolved from serological (method based on testing the reactivity of specific anti-sera with antigens) to molecular typing involving all HLA Class I and II alleles in the last decade. The latter, combined with advances in structural HLA modelling, have provided opportunities for more accurate assessment of HLA compatibility at the molecular level and underpinned an interest into defining the structural determinants of HLA allorecognition, also known as HLA B-cell epitopes. These epitopes consist of configurations of polymorphic amino acid residues expressed on HLA molecules that are recognized by the host’s immune system, generating an immune response that leads to the production of anti-HLA antibody.

The most commonly used algorithm for determining HLA compatibility at the molecular level is HLAMatchmaker which assumes that each HLA incorporates multiple structural epitopes (15-22 polymorphic amino acid residues) that form part of the binding surface with alloantibody, with each structural epitope encompassing at least one, smaller, “functional epitope” (cluster of surface-exposed amino acid residues at least one of which is polymorphic) called eplet, which determines the specificity and binding strength of the alloantibody-HLA interaction. Eplets comprise of short sequences of amino acid residues within a 3 Angstrom radius that interact directly with the paratope of an anti-HLA antibody (8, 9). HLA immunogenicity defined at the eplet level, expressed as the total number of eplet mismatches present in a donor HLA molecule, has been shown to provide a more accurate metric of HLA incompatibility beyond conventional broad antigen HLA mismatch with incremental eplet mismatches associated with an excess risk of acute antibody mediated rejection (AMR), development of dnDSA, transplant glomerulopathy and premature allograft loss in pediatric and adult kidney transplant recipients (9–17). Therefore, immunological risk assessment focused on quantifying the total number of eplet mismatches, calculated using HLAMatchmaker (18), may provide a more precise determination of immunological compatibility and subsequent risk stratification (8, 19). There are increasing data suggesting that the relationship between the number of eplet mismatches and adverse allograft outcome is not linear, and the idea of considering a population data-driven defined threshold, ignoring the relative immunogenicity of individual eplet mismatches, to inform clinical risk prediction is conceivably flawed. Several groups have attempted to provide insights into the differential and potentially hierarchical effects of individual or clusters of eplet mismatches (and at specific loci such as HLA-DQ), identification of high-risk eplet mismatches, and definition of eplet mismatch at the single HLA molecule level using novel machine learning statistical techniques that may provide a more accurate assessment of immunological risk (20–23).

Nevertheless, clinicians should be cognizant of the limitations of these studies and should cautiously interpret the study findings and applicability in clinical practice. Many of the studies reporting on the association between eplet mismatches and allograft outcome were confined to predominantly homogenous White populations, often undertaking imputation of the most likely 2-field molecular typing based on serological or low/intermediate resolution molecular typing using National Marrow Donor Program algorithm, the catalogue of Common and Well Documented (CWD) alleles integrated into the Allele Frequencies Net Database (AFND), Haplostats and the IMGT/HLA Database (24–27); and therefore misclassification bias of the total number and specificity of eplet mismatches may occur (28–32). The lack of large-scale data analysis from non-White populations, the inadequacies of imputation programs and presence of novel HLA alleles in Indigenous populations have further complicated the widespread acceptance of structural HLA compatibility in organ allocation and immunological risk assessment. There are several papers in this article collection that highlight the importance and limitations when considering HLA and non-HLA immunity in kidney transplantation. The paper by Larkins et al. highlights one of these issues and showed that a lack of high-resolution donor typing at the time of deceased donor kidney allocation can erroneously identify donor-specific anti-HLA antibody and eplet mismatches, which may have corresponding downstream effects on organ allocation and acceptance.

There is considerable debate regarding the optimal assessment of the immunogenicity of mismatched HLA alleles that could lead to the development of dnDSA and rejection. The methodology of defining clinically relevant immunogenic eplet mismatches and their reporting in the HLA-eplet registry is a subject of ongoing discussion (20, 33–35). The papers by Bezstarosti et al. review the evaluation of antibody-verified eplets, highlighting the need for internationally accepted standardized eplet verification methods. Moreover, using recombinant human HLA-DQ-specific monoclonal antibodies generated by isolated allospecific memory B cells from immunized individuals, the authors also propose a new platform for future antibody-verification of eplets within HLA-DQ alleles. Undoubtedly, additional studies verifying the risks associated with mismatched eplets captured in the HLA Eplet Registry in other well characterized cohorts are urgently required to validate the HLAMatchmaker eplet concept and potentially inform clinical care. It is important to note additional strategies and available software to assess donor/recipient HLA compatibility considering quantification of amino acid sequence polymopshism, differences in donor-recipient HLA physicochemical properties (3-dimensional electrostatic mismatch), and donor-derived Predicted Indirectly Recognizable HLA Epitopes presented by recipient HLA class II (PIRCHE-II, assesses the HLA-mismatch derived T-cell epitopes by quantifying the number of polymorphic donor HLA-derived peptides that can be presented on recipient HLA class II molecules). In addition to HLAMatchmaker, these algorithms have also been shown to predict adverse allograft outcomes post-kidney and simultaneous pancreas-kidney transplantation (36–45). Although these alternative algorithms independently predict the development of dnDSA beyond conventional broad antigen HLA-mismatches, there is little evidence to suggest that one algorithm is superior to another and a high degree of correlation between different outputs is often notable (46). Accordingly, the added advantage of a global integration of these measures in predicting HLA immunogenicity remains uncertain. Interestingly, a recent cohort study of 691 live-donor kidney transplant recipients suggested that eplet mismatches and PIRCHE score may be complementary in improving the discrimination of dnDSA risk, suggesting that a combined immunological risk prediction considering both B and T cell epitopes may be the way forward (43). Another European study showed that PIRCHE-II score may help to identify acceptable HLA mismatches that are associated with a lower risk of dnDSA independent of antigen mismatch and HLAMatchmaker epitopes, further suggesting the potential clinical applicability of this measure (40). With a growing body of evidence showing that mismatches at non-HLA variants may influence kidney transplant outcomes, a greater understanding of the clinical relevance of non-HLA genetic loci and their interaction with HLA-matching is required. The paper by Jethwani et al. provides a comprehensive review of the potential role and applicability of mismatches at non-HLA gene variants in predicting kidney allograft outcomes. The questions of the expected “value-added” of non-HLA variants in improving discrimination of adverse allograft outcomes above and beyond HLA incompatibility, as well as when and how to incorporate non-HLA genetic assessments in organ allocation and acceptance, require further study. Importantly, balancing between the cost-benefit of such approach must also be ensured. A group of younger kidney transplant candidates, who are more likely to require retransplantation in the context of suboptimal compatibility with their donors, may be the ideal group to benefit from a more precise assessment of HLA and non-HLA gene profiles.

Even though there are important caveats when considering the clinical applicability of utilizing structural HLA compatibility in immunological risk assessment and to inform allocation practices, there have been reports of successful integration of molecular compatibility in programs like the Eurotransplant Acceptable Mismatch program and in the context of deceased donor allocation to pediatric patients with kidney failure in Australia (47, 48). In a simulated model of implementing an alternative allocation strategy to avoid immunogenic “risk” molecular mismatches (i.e. mismatches associated with high risk of dnDSA) in lung transplantation, the avoidance of these high-risk mismatches could reduce the absolute rate of developing class II dnDSA by 30% (from 36% to 6%), with the trade-off that between 60% and 98% of donors would be excluded (22). On a practical level, by virtue of the population composition and more specifically the genetic profile of the donor pool and transplant candidates, different high risk molecular incompatibilities may be observed. Additionally, whether the added waiting time when striving to avoid high-risk mismatches at a population level might abolish the potential immunological gain from the avoidance of these high-risk molecular mismatches must be considered.

Despite the decreased incidence of acute rejection over the last few decades, this remains an important cause of allograft loss after kidney transplantation. Early identification and treatment of acute rejection is critical to avoid the deleterious effect on long-term allograft survival (49). Pre-transplant immunological risk stratification strategies can be used to personalize transplant immunosuppression and monitoring approaches and can be complemented by a careful assessment of the effect of immunosuppressive agents on the functionality, distribution and compartmentalization (peripheral blood, lymphoid organs, allograft) of alloreactive T and B cell subsets. This is critical to allow for ensuing strategies to personalized immunosuppression, balancing between the risk of “inadequate” immunosuppression (causing acute rejection) versus “excessive” immunosuppression (causing infection and cancer). While many centres administer induction immunosuppression based on patient’s sensitization history, the paper by Aschauer et al. reports that while reduced dose T-cell depleting induction therapy provided a greater early reduction of donor-reactive T cells compared to interleukin-2 inhibitor induction, there was no change on the overall T-cell receptor repertoire after immune reconstitution. The clinical implications of this observation and how the monitoring of circulating donor-specific T and B cells and corresponding T and B cell repertoires should inform modifications to immunosuppression regimens remain unknown and require evaluation in large prospective cohort studies and clinical trials.

The deleterious effect of post-transplant blood transfusion on risk of allosensitization (with development of dnDSA), acute rejection and allograft survival has been inconclusive, with the risk of allosensitization following blood transfusion being as high as 20% (50–55). However, the paper in this article collection by Jouve et al. challenged this notion by showing that blood transfusion in the first 3 months post-kidney transplant was not associated with an excess risk of developing dnDSA, with over 80% of these recipients receiving T-cell depleting antibody as induction therapy. Given the short-term follow-up of this study, the longer-term risk of allo-sensitization cannot be determined with certainty.

With the expansion in the knowledge of defining immunological risk and a greater availability and accessibility of high resolution molecular HLA typing techniques and Luminex technology to detect donor-specific anti-HLA and non-HLA antibodies, there continues to be a high degree of uncertainty as to the practical utility of eplet and other algorithms assessing structural HLA-compatibility, as well as the integration of this information into the complex process of organ allocation and decision making in clinical kidney transplantation. There is currently no single assay or measure that can capture all aspects of alloreactive cellular and humoral immune responses and the selection of one or multiple immunological risk prediction tools that may be complementary (or mutually exclusive) in risk stratification remain inadequately defined. Since the early seminal papers that have highlighted the potential clinical importance of HLA compatibility at the eplet or amino acid level, along with the prognostic significance of donor-specific anti-HLA and non-HLA antibodies, there has been an upsurge of studies that have attempted to validate these findings in different population cohorts and to identify other novel aspects of these associations or to report on diagnostic accuracy using big data and novel data science methodologies. However, there are important caveats the readers will need to consider when interpreting the study design, findings and conclusions of the growing number of publications addressing this issue, including highly selected and often homogenous patient populations and small sample sizes, differences in statistical techniques and adjustment of important confounders in multivariable models, lack of adherence to the Standards for Reporting of Diagnostic Accuracy Studies (STARD) statement (for diagnostic accuracy studies), inconsistent definitions and measurements of the predictors and outcomes, and the potential for inaccurate ascertainment of antibody profiles (incorrect or uncertain HLA imputation methods, lack of high resoluting typing across all HLA alleles and antibody verification) (56, 57). We hope this editorial, and selection of our article collection provides some insights into the challenges and limitations of the current landscape in the understanding of allo-immunity in kidney transplantation. We do envisage future prospective cohort studies and clinical trials focusing on a “global” evaluation of immunologic risk related to HLA and non-HLA-related injury and on immune monitoring using T cell receptor and other novel measures, such as HLA-specific B cells and donor-derived cell-free deoxyribonucleic acid, to provide much needed insight into this complex research field. Integration of the findings of such studies into clinical practice may inform future personalized immunosuppression strategies, reduce the risk of post-transplant allo-immune responses, and prolong allograft survival and clinical outcomes following kidney transplantation.

## Author contributions

All authors listed have made a substantial, direct, and intellectual contribution to the work and approved it for publication.

## Conflict of interest

The authors declare that the research was conducted in the absence of any commercial or financial relationships that could be construed as a potential conflict of interest.

## Publisher’s note

All claims expressed in this article are solely those of the authors and do not necessarily represent those of their affiliated organizations, or those of the publisher, the editors and the reviewers. Any product that may be evaluated in this article, or claim that may be made by its manufacturer, is not guaranteed or endorsed by the publisher.
